# Trends in cigarette consumption across the United States, with projections to 2035

**DOI:** 10.1371/journal.pone.0282893

**Published:** 2023-03-13

**Authors:** Eric C. Leas, Dennis R. Trinidad, John P. Pierce, Sara B. McMenamin, Karen Messer

**Affiliations:** Herbert Wertheim School of Public Health and Human Longevity Science, University of California San Diego, La Jolla, California, United States of America; New York University Grossman School of Medicine, UNITED STATES

## Abstract

**Objectives:**

To make projections of cigarette consumption that incorporate state-specific trends in smoking behaviors, assess the potential for states to reach an ideal target, and identify State-specific targets for cigarette consumption.

**Methods:**

We used 70 years (1950–2020) of annual state-specific estimates of per capita cigarette consumption (expressed as packs per capita or “ppc”) from the Tax Burden on Tobacco reports (N = 3550). We summarized trends within each state by linear regression models and the variation in rates across states by the Gini coefficient. Autoregressive Integrated Moving Average (ARIMA) models were used to make state-specific forecasts of ppc from 2021 through 2035.

**Results:**

Since 1980, the average rate of decline in US per capita cigarette consumption was 3.3% per year, but rates of decline varied considerably across US states (SD = 1.1% per year). The Gini coefficient showed growing inequity in cigarette consumption across US states. After reaching its lowest level in 1984 (Gini = 0.09), the Gini coefficient began increasing by 2.8% (95% CI: 2.5%, 3.1%) per year from 1985 to 2020 and is projected to continue to increase by 48.1% (95% PI = 35.3%, 64.2%) from 2020 to 2035 (Gini = 0.35; 95% PI: 0.32, 0.39). Forecasts from ARIMA models suggested that only 12 states have a realistic chance (≥50%) of reaching very low levels of per capita cigarette consumption (≤13 ppc) by 2035, but that all US states have opportunity to make some progress.

**Conclusion:**

While ideal targets may be out of reach for most US states within the next decade, every US state has the potential to lower its per capita cigarette consumption, and our identification of more realistic targets may provide a helpful incentive.

## Introduction

Trends in cigarette smoking prevalence have been projected forward with the suggestion that cigarette smoking might be entirely eliminated in the United States (US) by 2035, if the linear trend that occurred during the years of the Obama administration is continued [1]. While from a public health perspective reaching zero prevalence might be considered the ideal, many tobacco control experts believe that this target may be unrealistic as it may require inordinate resources or a comprehensive ban on cigarettes, neither of which may be possible in many countries including in the US [2]. Simple linear projections of national trends in cigarette smoking also make an assumption of “linear” progress; however, behaviors typically follow a more asymptotic or “S” shape curve–where they slowly approach low levels over many years [3]. Additionally, national targets may provide an overly optimistic outlook for many individual US states. For instance, while the US national cigarette smoking prevalence was 15.5% in the 2020 Behavioral Risk Factor Surveillance System (BRFSS) survey, in that same year state-level smoking prevalence varied from a high of 23.8% in West Virginia to a low of 7.9% in Utah [4, 5].

To set targets for the next decade, most jurisdictions have established thresholds below which they desire to reduce tobacco use and/or cigarette smoking prevalence. For example, in the US, Healthy People 2030 has set targets of achieving less than 5% prevalence of cigarette smoking [6] and less than 16.2% prevalence of any tobacco use, by 2030. Canada has set a more aggressive target of achieving less than 5% prevalence of any tobacco use in the general population of adults by 2035 [7, 8], and some Canadian provinces have adopted their own targets, including British Columbia and Quebec (each ≤10% cigarette smoking prevalence by 2023 [9, 10]). Australia has proposed targets of daily cigarette smoking prevalence of less than 10% by 2025, and less than 5% by 2030 [11]. The most aggressive targets to date are those in Finland (≤2% tobacco use by 2030 [12]), Ireland (≤5% cigarette smoking prevalence by 2025 [13]) and New Zealand (≤5% cigarette smoking prevalence by 2025 [14]). Although the overall goals of reducing cigarette smoking in the US have been set by Healthy People 2030, to our knowledge there has been only one quantitative assessment on a state-by state basis of the likelihood of reaching such a target, which relied on models using 10 years of state-level smoking prevalence data [5].

In this manuscript, our objective was to make projections of cigarette consumption that incorporate state-specific trends in smoking behaviors. To do so, we use Autoregressive Integrated Moving Average (ARIMA) models to project trends in state level per capita cigarette smoking, using 70 years of state-specific cigarette sales data. First, we describe trends in cigarette consumption for each US state, for the years 1950 through 2020. We illustrate how divergence in consumption rates between states has led to growing inequity in cigarette consumption across the US, as measured by the Gini coefficient [15], and highlight three US states that demonstrate the varied progress seen over the last 4 decades (California, Michigan, and Missouri). Next, we use the fitted ARIMA models to make annual state-specific forecasts of cigarette consumption, from 2021 through 2035. We use these state-specific forecasts to project the Gini coefficient in each year, to illustrate how unevenly distributed cigarette consumption may become across the US. We also use the state-specific forecasts to estimate each state’s “chance” of reaching an idealized target cigarette consumption in 2030 and in 2035, at a level chosen to approximate the Healthy People 2030 goal of ≤5% cigarette smoking prevalence, using simulated forecasts. For each state, we calibrated the chosen consumption target using the BRFSS, which includes prevalence data for each state for the last decade. Lastly, we used the state-specific forecasts to identify targets that we believe are “realistic” (estimated 50% chance of attainment) and “aggressive” (estimated 25% chance of attainment) for every US state.

## Methods

### Data sources

#### Cigarette sales data

Our sample consisted of annual estimates of per capita cigarette consumption (expressed as the number of tax-paid cigarette packs per capita or “ppc”) for each of the 50 US states (N = 3,550 total estimates of cigarette consumption), compiled from the 1950–2020 Tax Burden on Tobacco Reports. The Tax Burden on Tobacco is an annual compendium on tobacco revenue and industry statistics that began in 1950 and from 1989 onward has been prepared by the consulting firm Orzechowski and Walker [16]. These volumes are produced from information obtained from tobacco tax administrators in the 50 states, as well as from the US Department of Treasury’s Alcohol and Tobacco Tax Trade Bureau. Data are reported on an annual basis and include federal and state-level information regarding taxes applied to the price of a pack of cigarettes. Tax-paid sales represent the number of packs in each state for which state excise taxes were paid–either through the purchase of stamps by a wholesaler/distributor or through the filing of a monthly return by the wholesaler/distributor. For tax-paid sales per capita, the number of tax-paid sales for the state is divided by the population for the state as estimated by the US Census Bureau for the relevant year. The population figures used for the states are Census Bureau estimates as of July 1 of the respective fiscal years. Data for cigarette consumption can be found in Table 11 of the Tax Burden on Tobacco Reports for years 1950-Present. The first year of available data for each state is presented in **Table A in S1 Appendix**.

#### Identification of state specific cigarette consumption targets which approximate 5% smoking prevalence

To identify a value of per capita cigarette consumption that approximates the 5% smoking prevalence target set by many jurisdictions, for each US state we compared estimates of ppc from the Tax Burden on Tobacco reports with estimates of current smoking prevalence for the same year from the 2011–2020 BRFSS survey [4]. For each state and year, the BRFSS survey defines current smoking prevalence as the estimated number of persons aged 18+ years who reported ever smoking at least 100 cigarettes and who currently smoked every day or on some days (excluding respondents who answered "don’t know" or who refused to answer the survey questions), divided by the estimated total number of persons aged 18+ years in the same state and year.

**Fig 1:**
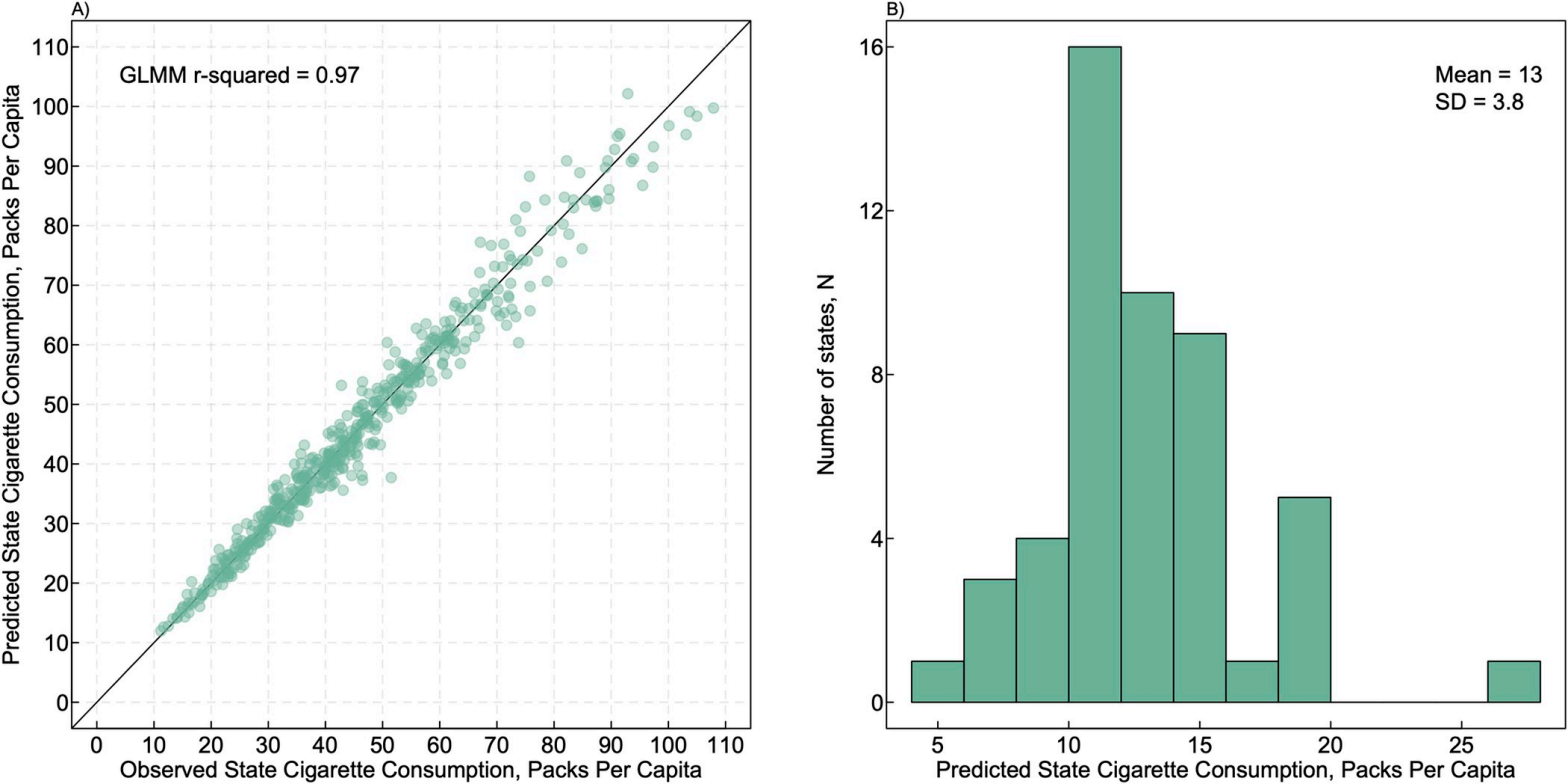
Observed and predicted cigarette consumption. **A**, Each dot represents observed cigarette consumption levels from 2011–2020 (x-axis) plotted against predicted consumption levels (y-axis) estimated from a model that included smoking prevalence estimates from the 2011–2020 Behavioral Risk Factor Surveillance System and a random effect for states (the model is shown in **Table B in S1 Appendix** and described in the methods). **B**, shows the distribution of values based on predictions from the model to 5% smoking prevalence for each of the 50 US states.

We used a random intercepts model to regress state-level ppc on adult cigarette smoking prevalence (**Table B in S1 Appendix**), using the *lme4* package in R. The model was $Y_{ij}=\mu +{\beta X}_{ij}+\alpha_{j}+\epsilon_{ij}$ where *i* indicates the year and *j* the US state, Y_ij_ represents log per capita cigarette consumption (ppc), μ the overall mean, X_ij,_ the log of state-level smoking prevalence, and where α_j_ is a mean-zero random intercept term for each state, and ε_ij_ residual mean-zero error. We log-transformed both cigarette consumption and smoking prevalence to enforce positivity of the fitted ppc values and to account for heteroscedasticity in the residual errors. A conditional LMM-R^2^ value [17] suggested that the model provided an excellent fit to the data, explaining 97% of the total variance in cigarette consumption (**Fig 1A**). We then used the fitted model to identify the estimated cigarette consumption (ppc, Y) for each state which corresponds to the idealized ≤5% smoking prevalence target (X) set by Healthy People 2030. We used the state-level estimated ppc values to obtain a corresponding population-average value of 13 ppc across US states (SD = 3.8 ppc) (**Fig 1B**). This suggested that a US population-level consumption of ≤13 ppc may provide a reasonable approximation to the idealized ≤5% smoking prevalence target, and we used this target in subsequent analyses.

### Statistical analysis

#### Summarizing historical trends in cigarette consumption

We summarized the annual change in cigarette consumption since 1980 for each state using linear regression to calculate a regression coefficient and corresponding 95% Confidence Interval (95% CI). The distribution of regression coefficients across states was summarized by the mean and standard deviation (SD).

#### ARIMA model selection and evaluation

For each US state, we forecast the expected per-capita cigarette consumption from 2021 through 2035 using a state-specific ARIMA model [18], reflective of the historical trends in per capita cigarette consumption. ARIMA models aim to improve over simpler regression models by describing the auto-correlation in the data as well as trends, and then incorporating this information into the projection [19]. An ARIMA model has three components: an autoregressive component of order *p*, a pre-processing component which differences the data of order *d*, and an error term given by a moving average of order *q*, expressed with the syntax ARIMA(*p*, *d*, *q*). We fit an ARIMA model to the consumption data for each state separately using the Hyndman-Khandakar algorithm, implemented using the auto.arima() function in the “forecast” package in R [20]. This algorithm combines unit root tests to determine the value of *d*, followed by minimization of the sample-size-adjusted Akaike Information Criterion to select values of *p* and *q*. We also allowed for potential inclusion of a “drift” constant. Consumption was modeled on the log scale. To assess model forecast accuracy, because our forecasts project 15 years into the future (2021–2035), we trained the models using data up to 2005 and then assessed their accuracy in forecasting values 15 years into the future (2006–2020). Accuracy was judged by the mean absolute error (MAE), which in our case is the average absolute difference between the forecast and observed values of annual per capita consumption from 2006 to 2020, for each state. We compared our ARIMA models to models fit on the same data using exponential smoothing models (ETS models), which use weighted averages of past observations with the weights decaying exponentially as the observations get older, as implemented using the ets() function in the “forecast” package in R [20]. On average, the ARIMA models appeared to be more accurate than the ETS models trained on the sample of ppc values up to 2005 available for each state (**Table C in S1 Appendix**). However, because cigarette consumption increased up to 1970 before beginning to decline after 1980 for most US states, we also assessed whether trimming the observations for the training model to 25 years of data from 1980 to 2005 further improved the accuracy of the ARIMA forecasts. The MAEs suggested that, on average across states, the ARIMA models continued to out-perform the ETS models on the time series trimmed from 1980, and were more accurate on average than the models trained to the entire observation period (**Table C in S1 Appendix**). For this reason, our final models were trained on estimates of cigarette consumption with a 25-year lag (i.e., from 1996 to 2020). The final ARIMA model specifications for our forecasts are presented in **Table D in S1 Appendix**.

#### Forecasting through 2035

We then used the ARIMA models to make forecasts of cigarette consumption from 2021 through 2035 for each state, using the *forecast* function from the forecast package [20]. The residual values from each state-specific model were resampled to simulate n = 1000 possible “future paths” that included the addition of error for new simulated observations as the time horizon increased, allowing for increasing uncertainty at future time points [21]. A sensitivity analysis increasing the number of resamples to n = 5000 suggested that n = 1000 provided a stable estimate (**Table E in S1 Appendix**). We summarize the predictions using the median and the 2.5^th^ and 97.5^th^ percentiles of the forecast paths, representing 95% prediction intervals (PIs). Because the idealized target is expressed in a desired direction (≤13 ppc), we used the proportion of occurrences of simulated results that were below the threshold ppc level as an indication of how “likely” the target was to occur, for each state in each year. Additionally, we took the values of ppc at the 25^th^ and 50^th^ percentiles of the simulated results as indication of “aggressive” and “realistic” targets for 2030 and 2035.

#### Summarizing the spread of average cigarette consumption across US states

We used the Gini coefficient to summarize the dispersion (i.e., “inequity”) of per capita cigarette consumption across US states, within each year [22, 23]. Gini coefficients describe inequity by summarizing the dispersion of a quantitative measure [15]. The Gini coefficient is computed as one-half the average absolute difference in per capita consumption, averaged over all pairs of states, divided by the mean per capita consumption averaged across all states. In our case, the values of the Gini coefficient can range from 0 (complete equity), to 1 (maximum inequity) since cigarette consumption cannot be negative. The Gini coefficient is a popular measure for summarizing inequity, but other common measures include the Theil index and the coefficient of variation [24], both of which were highly correlated with the Gini coefficients across years (Pearson’s r >0.98). Any states without observations in the 1950s and 1960s were omitted from the calculations in those years. We summarized the annual change in the Gini coefficient after reaching its historical low point (1984) using linear regression. We computed the Gini coefficient across states for each forecast year from 2021 to 2035, using the n = 1000 simulated future paths in each year from the ARIMA models for each state, forming a distribution of n = 1000 possible Gini values for each forecast year. We summarize the predicted distribution of Gini values using the median and 95% PI for each year.

## Results

**Fig 2:**
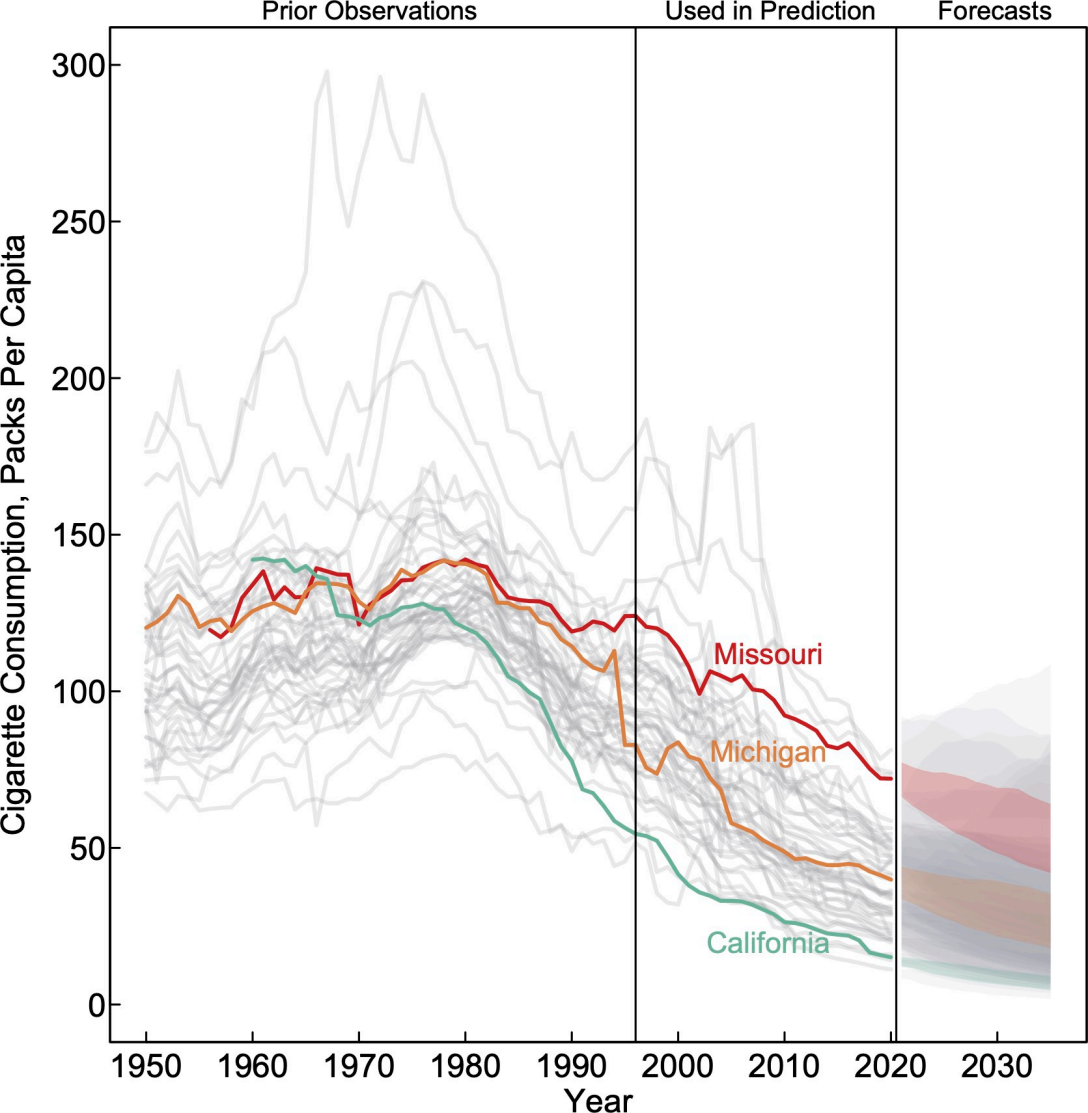
Trends in cigarette consumption across US states. After reaching their peak in the 1970s, cigarette consumption levels typically declined among US states from 1980 through 2020 and are expected to continue to decline for most US states. California, Michigan, and Missouri are highlighted as states that experienced diverging trends; all other state trends are shown in grey. Note: the forecasts are based on autoregressive integrated moving average models selected by Hyndman and Khandakar’s model selection algorithm and historical trends for each state with data for each forecast based on estimates from 1996 to 2020.

Rates of cigarette consumption typically grew in the 1950s and 1960s before reaching their peak in the 1970s and beginning to decline in the 1980s (**Fig 2**). Since 1980, the average rate of decline in cigarette consumption across US states was -3.3% per year, but rates varied considerably (SD = 1.1% per year) between US states (**Table 1**). For example, although California (120.2 ppc), Michigan (140.7 ppc) and Missouri (142.1 ppc) had very high consumption levels in 1980, the annual rate of decline in cigarette consumption in California (-5.2% per year; 95% CI = -5.4, -5.0) was 1.5 times the rate of decline in Michigan (-3.5% per year; 95% CI = -3.7, -3.3) and 3.3 times the rate of decline in Missouri (-1.6% per year; 95% CI = -1.7, -1.4). Consequently, by 2020 the level of cigarette consumption in California (15.1 ppc) was 2.6 times lower than the level of consumption in Michigan (39.9 ppc) and 4.8 times lower than the level consumption in Missouri (72.1 ppc).

**Table 1:** Log-linear regressions summarizing the annual trends in cigarette consumption for US states from 1980 to 2020.

(Sorted by coefficient size)	1980 ppc	Coefficient (95% CI)
New York	127.6	-6.7 (-7.1, -6.4)
Washington	101.4	-5.5 (-5.8, -5.2)
California	120.2	-5.2 (-5.4, -5.0)
Arizona	126.8	-5.0 (-5.3, -4.8)
Massachusetts	120.5	-4.8 (-5.0, -4.6)
New Jersey	124.3	-4.8 (-5.0, -4.5)
Maryland	131.5	-4.6 (-4.7, -4.4)
Rhode Island	149.3	-4.5 (-4.8, -4.3)
Nevada	177.7	-4.4 (-4.7, -4.2)
Vermont	161.6	-4.4 (-4.7, -4.1)
Connecticut	118.0	-4.4 (-4.6, -4.1)
Illinois	135.2	-4.3 (-4.5, -4.0)
New Mexico	102.7	-4.2 (-4.5, -4.0)
Alaska	134.0	-4.1 (-4.3, -3.9)
Utah	74.8	-4.0 (-4.3, -3.7)
Texas	129.7	-4.0 (-4.1, -3.8)
Minnesota	117.7	-3.9 (-4.3, -3.5)
Colorado	131.0	-3.8 (-4.0, -3.6)
Kansas	127.1	-3.7 (-3.9, -3.5)
Oregon	146.5	-3.7 (-3.9, -3.6)
Michigan	140.7	-3.5 (-3.7, -3.3)
North Carolina	187.8	-3.4 (-3.6, -3.2)
Florida	139.0	-3.4 (-3.7, -3.2)
Maine	141.2	-3.3 (-3.5, -3.2)
Georgia	134.0	-3.2 (-3.4, -3.0)
Wisconsin	117.6	-3.2 (-3.5, -2.9)
Pennsylvania	124.0	-3.1 (-3.3, -2.8)
Montana	122.0	-3.1 (-3.3, -2.8)
South Dakota	114.7	-3.0 (-3.3, -2.7)
Idaho	115.2	-3.0 (-3.2, -2.8)
Ohio	133.5	-3.0 (-3.2, -2.7)
Hawaii	89.4	-2.9 (-3.3, -2.5)
Wyoming	158.1	-2.8 (-3.1, -2.5)
Iowa	124.6	-2.8 (-3.1, -2.5)
Arkansas	131.8	-2.7 (-3.0, -2.5)
Nebraska	116.3	-2.7 (-2.8, -2.6)
New Hampshire	247.8	-2.7 (-2.9, -2.4)
Indiana	146.9	-2.6 (-2.9, -2.3)
Virginia	148.9	-2.6 (-2.7, -2.4)
South Carolina	138.3	-2.5 (-2.8, -2.2)
Kentucky	215.3	-2.4 (-2.8, -2.0)
Tennessee	130.4	-2.3 (-2.6, -2.0)
Louisiana	143.8	-2.3 (-2.5, -2.0)
Oklahoma	141.6	-2.2 (-2.6, -1.8)
Alabama	123.2	-2.1 (-2.3, -1.9)
Mississippi	127.0	-2.0 (-2.3, -1.8)
Delaware	150.5	-1.9 (-2.6, -1.3)
Missouri	142.1	-1.6 (-1.7, -1.4)
North Dakota	123.7	-1.4 (-1.7, -1.1)
West Virginia	122.3	-0.6 (-0.9, -0.4)

ppc = packs per capita; Note: coefficient values are the annual percent change in cigarette consumption

In **Table 2**, we list the p-value expressing each state’s chance of reducing their consumption below the target of 13ppc in 2030 and 2035. The forecasts from the ARIMA models suggested that eight states (California, New York, Utah, Washington, Massachusetts, Connecticut, Arizona, and New Mexico) have at least a 50% chance of reaching the 13 ppc target by 2030 and an additional four states (Illinois, New Jersey, Minnesota, and Maryland) have at least a 50% chance of reaching this target by 2035. However, the forecasts also suggest that 22 US states have essentially no chance (<1%) of reaching 13 ppc by 2035 and an additional 7 states have a less than a 5% chance of reaching 13 ppc by 2035.

**Table 2:** Probability of reaching idealized target cigarette consumption by 2030 and 2035 for US states.

State	2030		2035	
(Sorted by 2030 probability then alphabetically)	Median (95% PI)	Pr(≤13 ppc)	Median (95% PI)	Pr(≤13 ppc)
California	9.0 (6.4–11.1)	1.00	6.8 (4.6–9.0)	1.00
New York	5.5 (3.1–8.1)	1.00	3.8 (1.9–6.3)	1.00
Utah	9.3 (7.0–11.7)	1.00	7.2 (5.0–9.7)	1.00
Washington	8.0 (5.5–10.8)	1.00	5.8 (3.8–8.8)	1.00
Massachusetts	11.5 (7.8–16.0)	0.81	8.7 (5.4–13.2)	0.98
Connecticut	12.3 (9.0–17.1)	0.72	9.5 (6.4–14.2)	0.96
Arizona	12.5 (8.9–17.5)	0.67	9.8 (6.4–15.1)	0.94
New Mexico	13.5 (6.5–18.1)	0.50	10.3 (4.8–16.0)	0.85
Illinois	14.3 (9.0–19.9)	0.39	11.2 (6.6–17.0)	0.80
New Jersey	14.3 (10.4–18.9)	0.37	11.2 (7.5–15.4)	0.87
Minnesota	14.6 (9.8–19.6)	0.34	11.2 (7.2–16.8)	0.83
Maryland	16.8 (10.9–21.9)	0.15	13.5 (8.4–19.1)	0.50
Hawaii	22.3 (9.0–64.9)	0.13	23.1 (6.3–79.4)	0.20
Rhode Island	18.5 (12.2–25.3)	0.06	14.8 (9.1–21.3)	0.35
Nevada	18.5 (12.8–25.9)	0.05	14.5 (9.2–21.8)	0.36
Vermont	18.5 (13.1–24.7)	0.04	14.4 (9.5–20.9)	0.38
Florida	25.0 (13.8–31.6)	0.02	20.1 (10.7–28.2)	0.11
Kansas	20.7 (13.9–26.0)	0.02	16.5 (10.6–22.4)	0.17
Iowa	31.3 (17.4–44.0)	0.01	26.5 (13.2–41.7)	0.03
Wisconsin	38.5 (16.7–67.8)	0.01	38.3 (13.5–83.3)	0.03
Texas	19.0 (15.1–23.5)	0.00	15.6 (11.5–21.0)	0.17
Delaware	36.3 (18.4–66.3)	0.00	30.2 (13.6–63.5)	0.03
Alaska	18.7 (15.2–22.2)	0.00	14.7 (11.4–18.5)	0.25
Montana	24.6 (17.4–30.9)	0.00	20.2 (13.5–27.5)	0.03
Ohio	32.5 (22.0–38.9)	0.00	26.9 (17.4–34.5)	0.00
Pennsylvania	23.4 (17.1–29.1)	0.00	19.1 (13.1–25.2)	0.03
Alabama	37.7 (32.5–46.6)	0.00	32.7 (26.6–42.4)	0.00
Arkansas	32.9 (26.8–38.6)	0.00	27.5 (21.2–33.7)	0.00
Colorado	18.9 (16.0–22.0)	0.00	15.2 (12.4–18.6)	0.11
Georgia	28.7 (24.3–33.1)	0.00	23.8 (19.6–28.0)	0.00
Idaho	24.8 (19.4–31.1)	0.00	21.2 (15.5–28.1)	0.00
Indiana	39.4 (25.8–52.4)	0.00	33.1 (19.9–48.2)	0.00
Kentucky	50.4 (31.5–85.3)	0.00	41.5 (24.1–77.6)	0.00
Louisiana	35.7 (27.0–47.8)	0.00	30.7 (21.5–43.1)	0.00
Maine	36.5 (24.6–52.3)	0.00	31.4 (19.4–51.0)	0.00
Michigan	29.5 (22.1–38.6)	0.00	25.3 (18.3–35.9)	0.00
Mississippi	42.6 (28.2–53.1)	0.00	36.7 (23.1–50.5)	0.00
Missouri	57.3 (48.2–69.5)	0.00	50.8 (41.5–64.6)	0.00
Nebraska	29.2 (24.5–35.3)	0.00	25.1 (20.4–31.6)	0.00
New Hampshire	62.4 (40.2–93.0)	0.00	54.1 (32.2–89.1)	0.00
North Carolina	33.6 (26.1–41.2)	0.00	27.5 (20.2–35.8)	0.00
North Dakota	70.1 (59.8–86.7)	0.00	69.7 (60.0–86.5)	0.00
Oklahoma	33.0 (19.2–45.8)	0.00	27.4 (14.9–42.2)	0.01
Oregon	21.8 (17.8–27.6)	0.00	17.4 (13.4–23.5)	0.03
South Carolina	34.6 (22.2–53.8)	0.00	29.4 (18.2–52.6)	0.00
South Dakota	24.9 (17.1–34.7)	0.00	20.5 (12.7–31.7)	0.04
Tennessee	38.6 (23.0–48.9)	0.00	32.6 (19.1–44.6)	0.00
Virginia	40.2 (29.5–54.0)	0.00	34.6 (24.7–49.1)	0.00
West Virginia	73.8 (51.1–100.5)	0.00	73.1 (47.3–109.1)	0.00
Wyoming	52.3 (25.5–74.1)	0.00	52.4 (23.3–81.6)	0.00

ppc = packs per capita; Note: Because the idealized target is expressed in a desired direction (≤13 ppc), we used the proportion of occurrences of simulated results that were below the threshold ppc level as an indication of how likely the target was to occur, for each state in each year.

In **Table 3**, we list the values of ppc at the 25^th^ and 50^th^ percentiles of the simulated results, as indication of “aggressive” and “realistic” target thresholds for 2030 and 2035 for each state based on the forecasts. For example, based on the forecasts, California has a realistic chance of seeing consumption fall below 9 ppc by 2030, and below 7 ppc by 2035; whereas these projected targets were 29 ppc by 2030 and 25 ppc by 2035 for Michigan and 57 ppc by 2030 and 51 ppc by 2035 for Missouri. The aggressive targets for California were 8 ppc by 2030 and 6 ppc by 2035, for Michigan these were 27 ppc by 2030 and 22 ppc by 2035, and for Missouri the target was 53 ppc by 2030 and 47 ppc by 2035.

**Table 3:** Aggressive and realistic cigarette consumption target thresholds for 2030 and 2035 for US states.

State	2030		2035	
(Sorted alphabetically)	Aggressive target ppc (≤25^th^ percentile)	Realistic target ppc (≤50^th^ percentile)	Aggressive target ppc (≤25^th^ percentile)	Realistic target ppc (≤50^th^ percentile)
Alabama	35	38	30	33
Alaska	17	19	14	15
Arizona	11	13	9	10
Arkansas	31	33	25	28
California	8	9	6	7
Colorado	18	19	14	15
Connecticut	11	12	8	9
Delaware	29	36	23	30
Florida	20	25	16	20
Georgia	27	29	22	24
Hawaii	17	22	15	23
Idaho	23	25	19	21
Illinois	12	14	9	11
Indiana	34	39	28	33
Iowa	26	31	22	27
Kansas	18	21	14	17
Kentucky	43	50	35	41
Louisiana	32	36	27	31
Maine	32	36	27	31
Maryland	15	17	11	14
Massachusetts	10	12	7	9
Michigan	27	29	23	25
Minnesota	13	15	10	11
Mississippi	37	43	32	37
Missouri	54	57	47	51
Montana	22	25	18	20
Nebraska	27	29	23	25
Nevada	16	19	13	14
New Hampshire	54	62	46	54
New Jersey	13	14	10	11
New Mexico	11	14	8	10
New York	5	5	3	4
North Carolina	31	34	25	27
North Dakota	65	70	66	70
Ohio	28	33	24	27
Oklahoma	28	33	23	27
Oregon	20	22	16	17
Pennsylvania	21	23	17	19
Rhode Island	16	19	13	15
South Carolina	31	35	25	29
South Dakota	22	25	17	21
Tennessee	33	39	27	33
Texas	17	19	14	16
Utah	9	9	6	7
Vermont	16	19	12	14
Virginia	37	40	31	35
Washington	7	8	5	6
West Virginia	65	74	65	73
Wisconsin	30	38	28	38
Wyoming	43	52	40	52

ppc = packs per capita per; Note: We took the values of ppc at the 25^th^ and 50^th^ percentiles of the simulated results as indication of “aggressive” and “realistic” target thresholds for 2030 and 2035.

**Fig 3:**
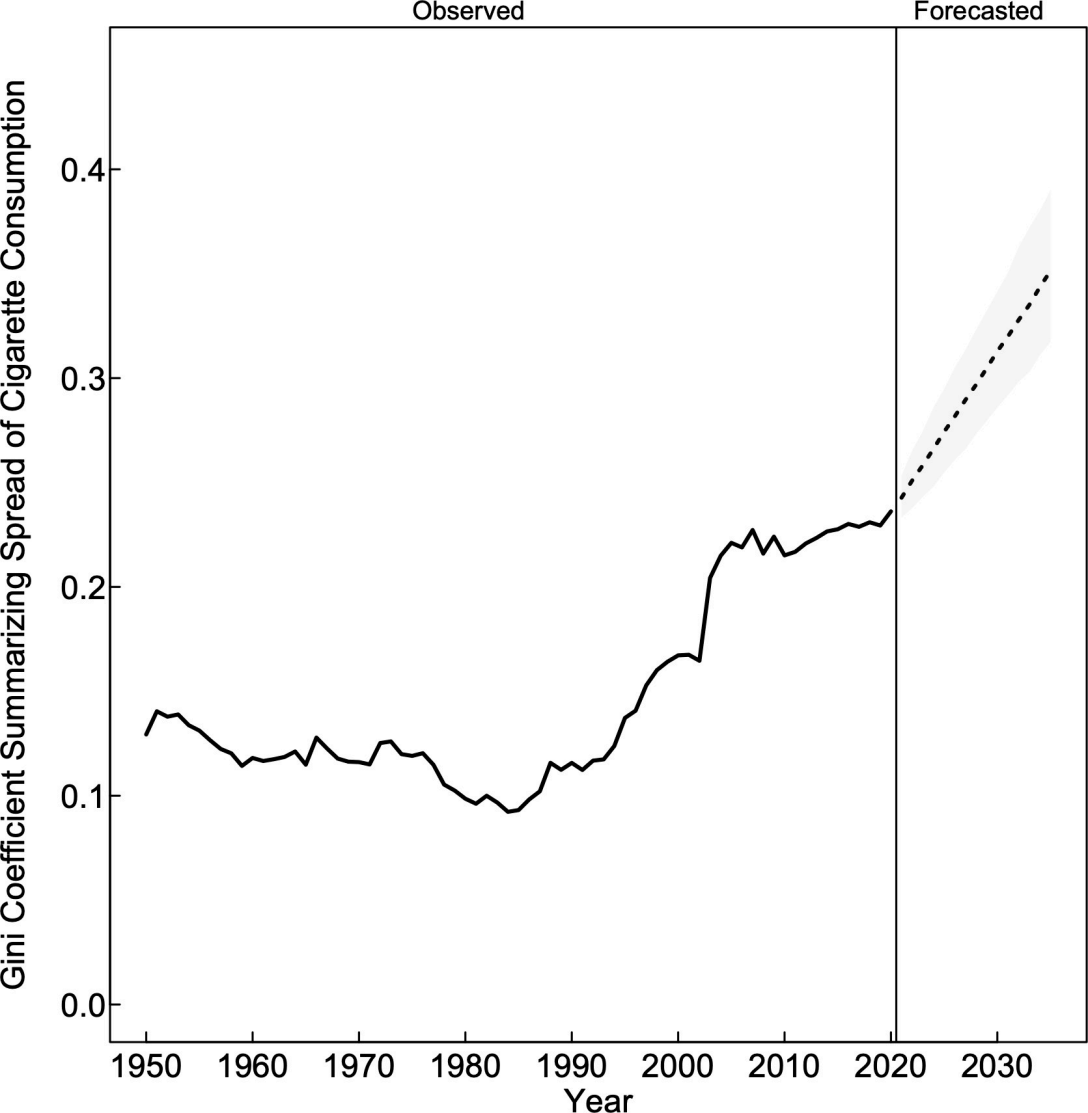
Inequity in cigarette consumption across US states. Overall declines in cigarette consumption from the mid-1980s onward were accompanied by growing inequality across US states, as measured by the Gini Coefficient. Inequality is expected to continue to grow if recent trends are maintained. Note: the forecasts are based on autoregressive integrated moving average models selected by Hyndman and Khandakar’s model selection algorithm and historical trends for each state with data for each forecast based on estimates from 1996 to 2020; Gini Coefficients are calculated within each year, across observed or forecast cigarette consumption for all available US states.

The diverging trends in cigarette consumption resulted in growing inequity across US states (**Fig 3**). After reaching the lowest level of inequity in 1984 (Gini = 0.09), inequity between US states began increasing by 2.8% per year (95% CI: 2.5, 3.1) from 1985 to 2020 (analysis not shown). If recent trends are maintained, the forecasts from the ARIMA models suggest that many US states are expected to continue to diverge in their consumption levels. For example, by 2035 the level of cigarette consumption in California (6.8 ppc; 95% PI = 4.6, 9.0) is expected to be 3.7 times lower than the level of consumption in Michigan (25.3 ppc; 95% PI = 17.6, 35.4) and 7.4 times lower than the level consumption in Missouri (50.8 ppc; 95% PI = 40.9, 65.0) (**Table 2**). As a result, inequity is expected to increase by 32.1% (95% PI = 21.2, 44.4) from 2020 (Gini = 0.24) to 2030 (Gini = 0.31; 95% PI: 0.29, 0.34) and by 48.1% (95% PI = 35.3, 64.2) from 2020 to 2035 (Gini = 0.35; 95% PI: 0.32, 0.39) (**Fig 3**).

## Discussion

Using 70 years of state-specific cigarette sales data, we have demonstrated that while state cigarette consumption has declined for all US states, it has done so at very different rates since the 1980s. These unequal rates of decline have led to growing inequity in cigarette consumption across US states, as measured by the Gini coefficient, and this inequity is expected continue to increase over the next 15 years. Our forecasts also suggest that, if recent trends continue, only a handful of states have a realistic chance of reducing per capita consumption to a level that reflects an idealized target, such as the ≤5% cigarette smoking prevalence target set by Healthy People 2030. However, progress is still possible for every US state, and we identify suggested targets that we believe are realistic for each state to pursue.

Comprehensive statewide tobacco control programs have been credited with the accelerated decline in per capita consumption observed in states such as California [25], New York [26], and Washington [27, 28], all of which are assessed as nearly certain to reach idealized targets by 2035. In California, effective tobacco control strategies have been in place long enough that they have also been linked to lower lung cancer rates [29, 30]. These aforementioned states have implemented many of the best practices outlined by both the US [31] and the World Health Organization’s MPOWER framework (monitor, protect, offer cessation, warn, enforcing advertising bans, and raise taxes) [32]. For example, each has substantially raised taxes, such that by 2022 the state excise tax on a pack cigarettes was $4.35 in New York, $3.02 in Washington, and $2.87 in California, compared to the average of $1.91 across all US states [33]. Each of these states has also implemented model smoke-free workplace policies which insure that 100% of indoor workers are protected from exposure [34]. Expanding coverage of such comprehensive statewide tobacco control strategies may help US states improve their chances of reaching the aggressive targets that we identified in our forecasts. In addition, we note that even successful comprehensive programs have opportunity for continued improvement. For example, states like Massachusetts have shown that new policy initiatives, such as comprehensive flavor bans, can lead to significant additional reductions in per capita cigarette consumption in states that already have successful programs [35].

In addition to state-run efforts, national and local efforts can also increase a state’s ability to close any gaps in policy coverage [36]. For example, an early key component of California’s tobacco control program involved supporting local coalitions to engage in public education and community mobilization, focused on influencing the local city council to implement a smoke-free workplace ordinance [37]. Similar local efforts focused on raising the minimum purchasing age to 21 years (“Tobacco 21” policies) through local ordinances provided sufficient evidence for the US Institute of Medicine to conclude in 2015 that raising the US minimum legal age for purchase of tobacco products to 21 would avert premature deaths [38], and by 2020 the US congress extended coverage of Tobacco 21 policies across all US states [39]. Such local initiatives may be a starting point in states that have lagging tobacco control efforts [40], and even when state or national policies exist, these policies can be further enhanced by local laws that ensure adequate enforcement [41].

A strength of our analyses was in the granularity of data available for cigarette consumption, with annual state-specific estimates of cigarette sales extending back to the 1950s for many US states. To our knowledge, this is the longest continuous time series from which it is possible to make state-specific forecasts of cigarette consumption. Another advantage is that The Tax Burden on Tobacco reports continue and are released annually, allowing for the tracking of progress, and updating of forecasts. There are also some limitations that should be considered. While our model suggests that prevalence and consumption are highly correlated (conditional LMM R^2^ = 0.97) in the available survey data from 2011–2020, the accuracy of our estimates for the “ideal target” depends on the extent to which this relationship is maintained from 2021 to 2035. Additionally, our estimates of the probability of achieving a target incorporate the estimated uncertainty in the forecasting model, so that higher model uncertainty can result in less certainty in target attainment. This can help explain why Maryland has a 50% chance of achieving the ideal target (13 ppc) at a median of 13.5 ppc, whereas Colorado has only an 11% chance at a median of 13.5 ppc. Further, as with most ARIMA models, our prediction interval estimates may be too narrow as only variation in the error term has been accounted for, however there is also uncertainty in the autocorrelation estimates and in the model order that has not been included in the forecasts. While the goal of our analysis was to identify targets based on historical trends, these sales trends may not be reliable for some states, especially those with significant illicit trade of cigarettes either entering or exiting the state [42] or where population structure may be changing in ways that are not reflected in the overall trend in cigarette consumption. Finally, while our analyses rely on a commonly used and well understood forecasting methodology, use the longest continuous data series available for US states, and followed best practices, future analyses could extend our models to provide estimates of the impacts of projected policy changes, such as sales tax changes [5], as well as test the approach against alternative forecasting strategies (e.g., models that co-integrate projections in both initiation and cessation) and datasets (e.g., surveys).

While acknowledging these limitations, our results suggest that most US states have essentially no chance of reaching aspirational national targets such as 5% smoking prevalence if the population-level trajectories from the last several decades continue to be followed. While ideal targets may be out of reach for most US states in the next decade, every US state has the potential to lower its cigarette consumption, and our identification of both more realistic and more aggressive state-specific targets may provide a helpful incentive. These results provide a strong call-to-action for expansion of tobacco control efforts in the US at national, state, and local levels.

## Supporting information

S1 Appendix Supplementary Appendix with additional supporting tables. (DOCX)
