# Supplementary material for: Trends in cigarette consumption across the United States, with projections to 2035
Source: PLoS One. 2023 Mar 13;18(3):e0282893. doi: 10.1371/journal.pone.0282893 (PMC10010542; doi:10.1371/journal.pone.0282893)
Supplement: S1 Appendix — (DOCX) [file pone.0282893.s001.docx]

**Table A. First year of data for each US state**

| State | First Year with Observations |
| --- | --- |
| (Sorted Alphabetically) | Year |
| Alabama | 1950 |
| Alaska | 1959 |
| Arizona | 1950 |
| Arkansas | 1950 |
| California | 1960 |
| Colorado | 1965 |
| Connecticut | 1950 |
| Delaware | 1950 |
| Florida | 1950 |
| Georgia | 1950 |
| Hawaii | 1960 |
| Idaho | 1950 |
| Illinois | 1950 |
| Indiana | 1950 |
| Iowa | 1950 |
| Kansas | 1950 |
| Kentucky | 1950 |
| Louisiana | 1950 |
| Maine | 1950 |
| Maryland | 1959 |
| Massachusetts | 1950 |
| Michigan | 1950 |
| Minnesota | 1950 |
| Mississippi | 1950 |
| Missouri | 1956 |
| Montana | 1950 |
| Nebraska | 1950 |
| Nevada | 1950 |
| New Hampshire | 1950 |
| New Jersey | 1950 |
| New Mexico | 1950 |
| New York | 1950 |
| North Carolina | 1970 |
| North Dakota | 1950 |
| Ohio | 1950 |
| Oklahoma | 1950 |
| Oregon | 1967 |
| Pennsylvania | 1950 |
| Rhode Island | 1950 |
| South Carolina | 1950 |
| South Dakota | 1950 |
| Tennessee | 1950 |
| Texas | 1950 |
| Utah | 1950 |
| Vermont | 1950 |
| Virginia | 1961 |
| Washington | 1950 |
| West Virginia | 1950 |
| Wisconsin | 1950 |
| Wyoming | 1952 |

**Table B. Fixed Effect for Smoking Prevalence from the Behavioral Risk Factor Surveillance System and Random Effects for States Resulting from the Generalized Linear Mixed Models for State Cigarette Consumption (n = 459).**

| *Fixed Effects* | *β* | *Standard Error* |
| --- | --- | --- |
| Intercept | 1.03 | 0.11 |
| Cigarette Consumption | 0.94 | 0.03 |
|  |  |  |
| *Random Effects* | *Variance* |  |
| Between-State | 0.090 |  |
| Residual | 0.007 |  |
|  |  |  |
| *Conditional GLMM-R^2^* | 0.97 |  |

Note: units are log scaled.

**Table C. Mean absolute error from 2006 to 2020 for ARIMA and ETS models trained on estimates of cigarette consumption from the entire observation period to 2005 or from 1980 to 2005**

| State | ETS trained on entire observation period to 2005 | ARIMA trained on entire observation period to 2005 | ETS trained from 1980 to 2005 | ARIMA trained from 1980 to 2005 |
| --- | --- | --- | --- | --- |
| (Sorted Alphabetically) | MAE | MAE | MAE | MAE |
| Alabama | 16.7 | 5.4 | 16.7 | 7.1 |
| Alaska | 20.2 | 20.2 | 20.2 | 7.0 |
| Arizona | 2.9 | 3.0 | 4.1 | 4.1 |
| Arkansas | 10.7 | 2.5 | 10.1 | 9.1 |
| California | 1.0 | 1.5 | 1.6 | 1.7 |
| Colorado | 1.6 | 4.0 | 5.3 | 5.3 |
| Connecticut | 2.2 | 2.1 | 6.4 | 5.0 |
| Delaware | 85.3 | 85.3 | 85.3 | 69.9 |
| Florida | 15.1 | 14.6 | 12.7 | 12.8 |
| Georgia | 1.4 | 3.8 | 4.8 | 3.9 |
| Hawaii | 15.5 | 15.5 | 15.5 | 15.5 |
| Idaho | 13.5 | 13.5 | 3.0 | 3.8 |
| Illinois | 6.3 | 2.2 | 2.6 | 4.0 |
| Indiana | 26.8 | 5.0 | 26.8 | 15.5 |
| Iowa | 31.9 | 31.9 | 30.3 | 21.2 |
| Kansas | 4.2 | 5.3 | 3.7 | 1.7 |
| Kentucky | 79.3 | 79.3 | 78.5 | 79.3 |
| Louisiana | 20.4 | 13.8 | 7.1 | 7.5 |
| Maine | 6.2 | 6.0 | 7.3 | 7.3 |
| Maryland | 1.8 | 1.7 | 1.9 | 2.4 |
| Massachusetts | 4.0 | 3.0 | 5.5 | 3.2 |
| Michigan | 4.1 | 5.2 | 2.3 | 2.9 |
| Minnesota | 28.6 | 17.9 | 17.3 | 17.4 |
| Mississippi | 19.5 | 12.3 | 13.0 | 12.9 |
| Missouri | 16.3 | 16.3 | 16.3 | 6.7 |
| Montana | 19.1 | 12.6 | 3.8 | 6.5 |
| Nebraska | 10.6 | 3.9 | 13.7 | 1.7 |
| Nevada | 7.6 | 7.5 | 7.3 | 7.0 |
| New Hampshire | 40.4 | 37.7 | 40.4 | 34.2 |
| New Jersey | 10.4 | 10.7 | 11.4 | 12.0 |
| New Mexico | 5.3 | 3.6 | 1.5 | 1.9 |
| New York | 2.2 | 1.9 | 2.1 | 1.8 |
| North Carolina | 28.0 | 20.1 | 13.7 | 13.9 |
| North Dakota | 6.7 | 6.3 | 7.2 | 16.6 |
| Ohio | 33.3 | 22.6 | 22.8 | 22.6 |
| Oklahoma | 24.6 | 35.6 | 30.5 | 28.2 |
| Oregon | 10.0 | 10.0 | 11.8 | 4.1 |
| Pennsylvania | 10.0 | 11.6 | 22.5 | 3.2 |
| Rhode Island | 6.9 | 8.5 | 18.5 | 2.5 |
| South Carolina | 26.1 | 26.2 | 26.2 | 26.2 |
| South Dakota | 23.1 | 23.1 | 13.3 | 13.1 |
| Tennessee | 29.1 | 22.2 | 29.2 | 20.5 |
| Texas | 14.0 | 6.9 | 5.4 | 5.5 |
| Utah | 10.8 | 7.4 | 3.7 | 3.7 |
| Vermont | 20.4 | 1.6 | 4.9 | 4.9 |
| Virginia | 3.3 | 4.7 | 6.1 | 2.8 |
| Washington | 3.3 | 3.0 | 5.1 | 5.2 |
| West Virginia | 12.0 | 16.2 | 12.1 | 14.2 |
| Wisconsin | 23.5 | 13.3 | 13.8 | 13.7 |
| Wyoming | 13.8 | 10.5 | 4.9 | 17.3 |
| **Mean** | **16.6** | **14.0** | **14.6** | **12.2** |

MAE = Mean Absolute Error; Entire observation period = estimates available from the first date of observations (listed in Supplementary Table 1) to 2005.

**Table D. Final ARIMA models fit to 1995–2020 estimates of cigarette consumption**

| State | Model |
| --- | --- |
| (Sorted Alphabetically) | ARIMA(p,d,q) |
| Alabama | ARIMA(0,1,0) with drift |
| Alaska | ARIMA(0,1,0) with drift |
| Arizona | ARIMA(0,1,0) with drift |
| Arkansas | ARIMA(1,1,0) with drift |
| California | ARIMA(0,1,0) with drift |
| Colorado | ARIMA(0,1,0) with drift |
| Connecticut | ARIMA(0,1,1) with drift |
| Delaware | ARIMA(0,1,0) with drift |
| Florida | ARIMA(0,1,0) with drift |
| Georgia | ARIMA(1,1,0) with drift |
| Hawaii | ARIMA(1,1,0) |
| Idaho | ARIMA(0,1,0) with drift |
| Illinois | ARIMA(1,1,0) with drift |
| Indiana | ARIMA(0,1,0) with drift |
| Iowa | ARIMA(0,1,1) with drift |
| Kansas | ARIMA(0,1,0) with drift |
| Kentucky | ARIMA(0,1,0) with drift |
| Louisiana | ARIMA(1,1,0) with drift |
| Maine | ARIMA(0,1,1) with drift |
| Maryland | ARIMA(0,1,0) with drift |
| Massachusetts | ARIMA(0,1,0) with drift |
| Michigan | ARIMA(0,1,0) with drift |
| Minnesota | ARIMA(1,1,0) with drift |
| Mississippi | ARIMA(0,1,0) with drift |
| Missouri | ARIMA(0,1,0) with drift |
| Montana | ARIMA(0,1,0) with drift |
| Nebraska | ARIMA(2,1,1) with drift |
| Nevada | ARIMA(1,1,0) with drift |
| New Hampshire | ARIMA(0,1,0) with drift |
| New Jersey | ARIMA(0,1,0) with drift |
| New Mexico | ARIMA(0,1,0) with drift |
| New York | ARIMA(0,1,0) with drift |
| North Carolina | ARIMA(0,1,0) with drift |
| North Dakota | ARIMA(2,0,0) |
| Ohio | ARIMA(0,1,0) with drift |
| Oklahoma | ARIMA(0,1,0) with drift |
| Oregon | ARIMA(0,1,0) with drift |
| Pennsylvania | ARIMA(0,1,0) with drift |
| Rhode Island | ARIMA(0,1,0) with drift |
| South Carolina | ARIMA(0,1,0) with drift |
| South Dakota | ARIMA(0,1,0) with drift |
| Tennessee | ARIMA(1,1,0) with drift |
| Texas | ARIMA(0,1,0) with drift |
| Utah | ARIMA(1,1,0) with drift |
| Vermont | ARIMA(0,1,0) with drift |
| Virginia | ARIMA(0,1,0) with drift |
| Washington | ARIMA(0,1,1) with drift |
| West Virginia | ARIMA(0,1,0) |
| Wisconsin | ARIMA(1,1,0) |
| Wyoming | ARIMA(0,1,1) |

**Table E. Sensitivity analyses comparing choice of n=1000 vs. n=5000 replications for bootstrap prediction intervals from final ARIMA models fit to 1995–2020 estimates of cigarette consumption**

|  | 2030 | | | 2035 | | |
| --- | --- | --- | --- | --- | --- | --- |
| State | n=1000 replicates | n=5000 replicates | | n=1000 replicates | n=5000 replicates | |
| (Sorted Alphabetically) | Median | Median | Difference | Median | Median | Difference |
| Alabama | 37.7 | 37.7 | 0.0 | 32.7 | 32.6 | 0.1 |
| Alaska | 18.7 | 18.6 | 0.1 | 14.7 | 14.7 | 0.0 |
| Arizona | 12.5 | 12.6 | 0.0 | 9.8 | 9.8 | 0.0 |
| Arkansas | 32.9 | 32.9 | 0.0 | 27.5 | 27.7 | -0.2 |
| California | 9.0 | 9.0 | 0.1 | 6.8 | 6.9 | 0.0 |
| Colorado | 18.9 | 19.0 | -0.1 | 15.2 | 15.2 | 0.0 |
| Connecticut | 12.3 | 12.3 | 0.0 | 9.5 | 9.5 | 0.0 |
| Delaware | 36.3 | 36.4 | -0.1 | 30.2 | 29.8 | 0.4 |
| Florida | 25.0 | 25.5 | -0.5 | 20.1 | 20.7 | -0.5 |
| Georgia | 28.7 | 28.7 | 0.0 | 23.8 | 23.7 | 0.1 |
| Hawaii | 22.3 | 22.2 | 0.1 | 23.1 | 22.6 | 0.5 |
| Idaho | 24.8 | 24.7 | 0.2 | 21.2 | 20.9 | 0.3 |
| Illinois | 14.3 | 14.1 | 0.2 | 11.2 | 10.9 | 0.3 |
| Indiana | 39.4 | 40.4 | -1.0 | 33.1 | 33.4 | -0.2 |
| Iowa | 31.3 | 31.4 | -0.1 | 26.5 | 26.5 | 0.0 |
| Kansas | 20.7 | 20.9 | -0.3 | 16.5 | 16.9 | -0.4 |
| Kentucky | 50.4 | 50.3 | 0.1 | 41.5 | 41.9 | -0.4 |
| Louisiana | 35.7 | 35.4 | 0.3 | 30.7 | 29.9 | 0.8 |
| Maine | 36.5 | 36.2 | 0.3 | 31.4 | 31.3 | 0.1 |
| Maryland | 16.8 | 16.7 | 0.1 | 13.5 | 13.3 | 0.2 |
| Massachusetts | 11.5 | 11.5 | 0.1 | 8.7 | 8.7 | 0.1 |
| Michigan | 29.5 | 29.2 | 0.3 | 25.3 | 25.1 | 0.2 |
| Minnesota | 14.6 | 14.4 | 0.2 | 11.2 | 11.1 | 0.1 |
| Mississippi | 42.6 | 42.1 | 0.5 | 36.7 | 36.7 | 0.1 |
| Missouri | 57.3 | 57.5 | -0.2 | 50.8 | 51.5 | -0.7 |
| Montana | 24.6 | 24.6 | 0.0 | 20.2 | 20.5 | -0.3 |
| Nebraska | 29.2 | 29.1 | 0.1 | 25.1 | 25.0 | 0.0 |
| Nevada | 18.5 | 18.9 | -0.3 | 14.5 | 14.6 | -0.2 |
| New Hampshire | 62.4 | 61.9 | 0.5 | 54.1 | 53.9 | 0.3 |
| New Jersey | 14.3 | 14.4 | -0.1 | 11.2 | 11.1 | 0.0 |
| New Mexico | 13.5 | 13.5 | 0.0 | 10.3 | 10.4 | -0.1 |
| New York | 5.5 | 5.5 | -0.1 | 3.8 | 3.9 | 0.0 |
| North Carolina | 33.6 | 33.6 | 0.0 | 27.5 | 27.6 | -0.2 |
| North Dakota | 70.1 | 69.7 | 0.4 | 69.7 | 69.8 | -0.1 |
| Ohio | 32.5 | 32.7 | -0.1 | 26.9 | 27.2 | -0.3 |
| Oklahoma | 33.0 | 33.8 | -0.8 | 27.4 | 27.8 | -0.4 |
| Oregon | 21.8 | 21.7 | 0.1 | 17.4 | 17.3 | 0.1 |
| Pennsylvania | 23.4 | 23.3 | 0.1 | 19.1 | 19.0 | 0.1 |
| Rhode Island | 18.5 | 18.6 | -0.1 | 14.8 | 14.8 | 0.0 |
| South Carolina | 34.6 | 34.4 | 0.2 | 29.4 | 29.3 | 0.1 |
| South Dakota | 24.9 | 24.6 | 0.3 | 20.5 | 20.5 | 0.0 |
| Tennessee | 38.6 | 38.7 | -0.1 | 32.6 | 32.5 | 0.0 |
| Texas | 19.0 | 19.2 | -0.2 | 15.6 | 15.7 | 0.0 |
| Utah | 9.3 | 9.3 | 0.0 | 7.2 | 7.2 | 0.0 |
| Vermont | 18.5 | 18.5 | 0.0 | 14.4 | 14.3 | 0.1 |
| Virginia | 40.2 | 40.3 | -0.1 | 34.6 | 35.0 | -0.3 |
| Washington | 8.0 | 7.7 | 0.2 | 5.8 | 5.8 | 0.0 |
| West Virginia | 73.8 | 74.8 | -1.0 | 73.1 | 76.1 | -3.0 |
| Wisconsin | 38.5 | 39.3 | -0.8 | 38.3 | 39.8 | -1.5 |
| Wyoming | 52.3 | 52.1 | 0.2 | 52.4 | 51.5 | 0.9 |
